# Radical Operations for Bursal Enlargements1Read at the Thirty-eighth Annual Convention of the American Veterinary Medical Assotion, Atlantic City, N. J., September, 1901.

**Published:** 1901-11

**Authors:** C. C. Lyford

**Affiliations:** Minneapolis, Minn.


### RADICAL OPERATIONS FOR BURSAL ENLARGEMENTS.[1]

By C. C. Lyford, B.S., M.D., D.V.S.,
MINNEAPOLIS, MINN.

Accident, with nature as an assistant, is often a forerunner of both medical and surgical science and success.

Few veterinarians who have been in practice for a quarter of a century have not had opportunities of seeing lameness and bursal enlargements cured or improved as a result of direct injury. In the horse the fetlocks are especially subjected to wounds and bruises during runaways and kicking fracases, and in many cases of this kind I have noticed beneficial results.

[1] Read at the Thirty-eighth Annual Convention of the American Veterinary Medical Assotion, Atlantic City, N. J., September, 1901.

Bursal enlargements have been long considered dangerous ground for surgical interference, especially so with young practitioners having an idea that the veterinarian is the whole thing and nature his enemy; but sooner or later he will learn that nature is his best friend, and that to consult her oftener and his fellow-practitioner and books less frequently would prove beneficial not only to himself and his patient but to the owner of the animal.

In considering the treatment of cases, I shall take them up not as any particular class, but as they appeared in my practice during the past sixteen years.

Case I., June, 1885, a brother of Billy Dayton, 2.27½, was sent me for treatment, he being a confirmed cripple from distention and enlargement of the bursæ of fetlocks on both front legs. I at once aspirated and injected tincture of iodine. This was followed with cooling lotions and bandages for two weeks. Then three successive blisters were applied at intervals of ten days. The swelling by this time had entirely disappeared, and the horse jogged apparently as well as ever, so was returned to his owner, Mr. Dayton, who lived in Wisconsin. For the next two months he was driven on the road preparatory to some fast work that he received in September and October. In November he was returned to me with bursæ as large and patient as lame as ever. I then decided upon opening parts above fetlock of both front legs, injecting tincture of iodine (U. S. P.), following daily with corrosive solution, 1 : 500 parts, dressing wound with marine lint saturated with listerine, parts being kept cold with wet bandages. This was continued for four weeks, at which time the wounds were entirely closed. I then followed with blisters of cantharides and biniodide of mercury, repeating every two weeks for six weeks. At the end of two months the horse was apparently in good shape and was returned home the last of January, 1886. He was immediately given road work and used for speeding on the ice. I received a report from the owner during the spring that he showed no signs of returning trouble, that he intended to put him on the track; but the owner died shortly after this, and the horse was lost sight of.

Case II., October, 1895.—Was called to see gray gelding belonging to D. Clapsidle. Previous history indicated enlargement of fetlock, two years' standing. Said horse had met with an injury ten days prior to my visit, caused by a cut under the fetlock opening the bursal sheath. Having been dressed and bandaged, the cut had healed, though so distended and painful that he could not rest his weight on the foot. I at once opened above fetlock on both sides, passing the seton through and dressing the parts with antiseptics and keeping cool as in the previous case. The horse was given a box-stall and yard to run in. Parts were dressed twice daily. Wound had closed at the end of three weeks, when a blister was applied. Two weeks later he was put at work on a heavy meat wagon, from which time he had not lost a day's work until the fall of 1900, when he died from other causes. To all appearence this leg was as good as the other, and had never gone lame from the time of treatment.

CASE III.—Brown gelding was presented for treatment July 14, 1899, at the Minnesota State Veterinary Medical Association Meeting, held at Faribault. Subject was as near as possible a duplicate of Case II., excepting no wound, parts being painful from distention of the sheath and bursæ, resulting from a severe sprain. I advised operating, though the majority of the members thought differently. The owner coincided with me, and I assisted Dr. S. D. Brimhall in opening and putting seton in place and dressing parts. The horse was sent home with instructions to move seton and dress leg with antiseptics and cooling lotions twice a day. The only report from the case was given by Dr. Hay, of Faribault, six months later, who said the treatment was too successful to be profitable, as the owner never even consulted him regarding the case, but the horse was at work, and as good as ever, and the leg as small as its fellow.

CASE IV., June 26, 1900.—Bay mare Judith, belonging to the Minnesota State Experimental Station, having been sent to my infirmary to be delivered, but before removal got cast in the stall and produced a large fat-capped hock, so I called Drs. Reynolds and Brimhall to assist in the operation, which consisted in casting, cleansing, and cocaining the parts. An opening was made at the lower part of the swelling from behind upward to within half an inch of the apex. The sac was then separated from its attachments and removed. Parts were cared for as in previous cases recorded, and were healing nicely until ten days later patient got cast in the stall the second time, causing the entire hock to swell to double its original size. Then I decided to place her in sling, where she was kept until August 4th, the parts being nicely healed, showing only a small scar about the size of a nickel.

CASE V., October 20, 1900.—Was called to see William K., 2.23$\frac{1}{4}$, belonging to J. W. Hull, Minneapolis. History of long standing trouble, with more than a year of lameness at intervals.

The part affected, the left front leg at fetlock; large bleb appearing on outer and upper side about the size of a turkey's egg. I at once opened parts and injected sheath with iodine. By opening the sac I found it consisted of a number of coats, indicating the result of several attacks of inflammation. I removed as much of the external wall of sac as possible, and at the point of the external excision a depression is now to be seen.

William K. has been in use since March 1st, and is now free from lameness, with leg firm, and does not swell from using. His treatment was as near as possible a repetition of previous cases.

Thorough-pins may be simple or complicated ; in the latter case they extend down the sheath on the inner side of the hock, following the tendon of perforans, or may be connected with bursa of tibia—tarsal articulation—in which case I would not recommend a radical operation, though I have often seen punctured wounds entering the true joint that were healed without causing stiffness.

Case VI.—I was consulted during the spring of 1891 by the fire department of Anoka, Minn., regarding one of their horses, which was afterward sent for treatment to the Experimental Station, St. Anthony Park, a photograph of which Dr. Reynolds has kindly presented for your consideration. This case does not differ materially from Case VII. and Case VIII., excepting the size being much larger, and confined to the upper part of the hock.

Case VII.—This case appeared July 12, 1901, at our State Veterinary meeting, Northfield, Minn., the subject being a black gelding, weighing about 1100 pounds, though greatly emaciated from overwork and lameness of both hind legs, more especially the right, at stifle, which with the enlargement of his left hock, made it difficult for him to stand. The stifle was cocained and fired, pyro-punctured work being done by Dr. J. P. Foster, of Selby, South Dakota. The hock was then cleansed, shaven, and cocained, and opened at two different places on the inner and one on the outer part of the hock, the swelling being more extensive on the inner and upper portion, following the sheath of the perforans tendon downward and backward to nearly two inches below the chestnut, the enlargement on the inner surface of the hock being something over nine inches in length, with a septum between upper and lower portions of sac, which prevented direct communication between the two parts. Having thoroughly opened and explored the different cavities, a 10 per cent. solution of tincture of iodine was used to swab the parts out. After dressing the parts with antiseptic solution of corrosive sublimate, 1 : 1000, covering the parts with compresses, the leg was done up as in previous cases. The case showed no indication of hemorrhage two hours later, when the meeting adjourned, the patient being left at the infirmary of Dr. K. J. McKenzie, whose later reports on the case are very interesting:

"*July* 18, 1901. The patient with bursal enlargements is pretty weak at present. I was away all day the day following the meeting. On my return found my man trying in every way to stop hemorrhage from the leg that you operated on. He bled considerably. That was last Friday night, and last night (the 17th) I came in from the country and found the stall flooded with blood from the other leg where he had been fired. I hustled a hypodermic and salt solution, and he braced up considerable. The wounds are in excellent condition, and there has been very little swelling. I think he would have gotten along finely had he not had to lose so much blood.

"31*st*. Our patient with bursal enlargement is doing better, and I have hopes of his recovery. I used the saline solution subcutaneously after the hemorrhage, and got some beautiful sloughing about the neck.

"*August* 21*st*. Yours asking about the patient with bursal enlargement came to hand, and will say he is going to make a live of it all right. The parts in which the sloughing took place are granulating nicely. I evidently used the solution too strong, without proper antiseptic precaution, merely ordinary cleanliness. I have been using a bichloride, 1 : 1000, and it is doing nicely. I feel that the sodium chloride saved his life, though it raised 'Cain' with his common integument. They say he is not as lame on either leg as he was."

CASE VIII.—This case is so nearly a duplicate of Case VI. that it needs very little consideration, besides being only about one-half the size of Case VI. The history points to about one year's standing. I was called to operate August 16, 1901, at which time I was assisted by Dr. Reynolds, both in operating on and photographing the leg. The subject was placed in slings on account of lameness induced by a bursal sore at the inner portion of the coronary band of same foot. After thoroughly opening the parts and injecting tincture of iodine, the seton was passed through the leg, and leg dressed as in previous cases. Since the operation I have examined the case about twice, August 20th and August 27th. On the first date the leg was badly swollen and discharging profusely. The latter swelling had disappeared, so that the parts were smaller than at the time of operation, and the discharge was but slight, though the sore on the foot was still troublesome, having spread one third way around the foot.

As to advice regarding operations of this kind, one should not be afraid of cutting freely and giving parts thorough drainage, and see that the wounds are kept sufficiently open, and for such a time as to insure complete healing of internal parts and destruction of the bursæ, which should heal by granulation before the wound is closed.

The bloodvessels being larger and more numerous on the inside of legs at both hock and fetlock, cutting may be done freely on the outside, being more guarded while operating on the inner side, but by no means consider either sufficient alone, as I have invariably seen it fail where the opening was on one side and the injections could not be forced through; hence I consider a seton almost indispensable both for drainage and keeping the parts open.

